# Supplementary material for: Heparin-based hydrogel scaffolding alters the transcriptomic profile and increases the chemoresistance of MDA-MB-231 triple-negative breast cancer cells
Source: Biomater Sci. 2020 Feb 13;8(10):2786–96. doi: 10.1039/c9bm01481k (PMC7497406; doi:10.1039/c9bm01481k)
Supplement: Supplementary file 2 [file BM-008-C9BM01481K-s002.zip › Supplementary File 4/HP-BhydrogelvControl/Pathways/my_analysis.Gsea.1545201079907/HALLMARK_IL2_STAT5_SIGNALING.html]

Details for gene set HALLMARK\_IL2\_STAT5\_SIGNALING[GSEA]

|  || Dataset | expr.class.cls#HYD\_versus\_CONTROL.class.cls#HYD\_versus\_CONTROL\_repos |
| Phenotype | class.cls#HYD\_versus\_CONTROL\_repos |
| Upregulated in class | CONTROL |
| GeneSet | HALLMARK\_IL2\_STAT5\_SIGNALING |
| Enrichment Score (ES) | -0.25169867 |
| Normalized Enrichment Score (NES) | -1.1814827 |
| Nominal p-value | 0.08866995 |
| FDR q-value | 0.16555697 |
| FWER p-Value | 0.807 |
Table: GSEA Results Summary

  

Fig 1: Enrichment plot: HALLMARK\_IL2\_STAT5\_SIGNALING      
 Profile of the Running ES Score & Positions of GeneSet Members on the Rank Ordered List

  

| PROBE | DESCRIPTION (from dataset) | GENE SYMBOL | GENE\_TITLE | RANK IN GENE LIST | RANK METRIC SCORE | RUNNING ES | CORE ENRICHMENT || 1 | AKAP2 | na |  |  | 316 | 2.646 | 0.0092 | No |
| 2 | LRIG1 | na |  |  | 449 | 2.415 | 0.0259 | No |
| 3 | MAFF | na |  |  | 475 | 2.376 | 0.0478 | No |
| 4 | MAP3K8 | na |  |  | 1059 | 1.833 | 0.0350 | No |
| 5 | ENO3 | na |  |  | 1277 | 1.702 | 0.0402 | No |
| 6 | GPR83 | na |  |  | 1681 | 1.498 | 0.0337 | No |
| 7 | NOP2 | na |  |  | 1860 | 1.420 | 0.0382 | No |
| 8 | GADD45B | na |  |  | 1972 | 1.375 | 0.0458 | No |
| 9 | AGER | na |  |  | 2542 | 1.160 | 0.0273 | No |
| 10 | MYO1C | na |  |  | 2814 | 1.081 | 0.0236 | No |
| 11 | MAP6 | na |  |  | 3387 | 0.942 | 0.0027 | No |
| 12 | NCS1 | na |  |  | 3516 | 0.913 | 0.0049 | No |
| 13 | IKZF4 | na |  |  | 3555 | 0.905 | 0.0118 | No |
| 14 | MAPKAPK2 | na |  |  | 3706 | 0.870 | 0.0124 | No |
| 15 | FURIN | na |  |  | 3731 | 0.863 | 0.0195 | No |
| 16 | FAH | na |  |  | 3746 | 0.859 | 0.0272 | No |
| 17 | DENND5A | na |  |  | 3761 | 0.856 | 0.0348 | No |
| 18 | MYC | na |  |  | 3771 | 0.854 | 0.0427 | No |
| 19 | PUS1 | na |  |  | 3780 | 0.853 | 0.0506 | No |
| 20 | BATF3 | na |  |  | 3881 | 0.837 | 0.0535 | No |
| 21 | SLC1A5 | na |  |  | 4248 | 0.762 | 0.0417 | No |
| 22 | UCK2 | na |  |  | 4278 | 0.756 | 0.0476 | No |
| 23 | CD83 | na |  |  | 4324 | 0.747 | 0.0525 | No |
| 24 | APLP1 | na |  |  | 4385 | 0.735 | 0.0565 | No |
| 25 | SLC29A2 | na |  |  | 4773 | 0.664 | 0.0427 | No |
| 26 | HIPK2 | na |  |  | 4827 | 0.654 | 0.0463 | No |
| 27 | BMP2 | na |  |  | 4891 | 0.643 | 0.0493 | No |
| 28 | RHOH | na |  |  | 5035 | 0.621 | 0.0478 | No |
| 29 | TLR7 | na |  |  | 5057 | 0.618 | 0.0527 | No |
| 30 | PTCH1 | na |  |  | 5058 | 0.618 | 0.0588 | No |
| 31 | LIF | na |  |  | 5094 | 0.614 | 0.0629 | No |
| 32 | RNH1 | na |  |  | 5104 | 0.613 | 0.0684 | No |
| 33 | RGS16 | na |  |  | 5502 | 0.552 | 0.0530 | No |
| 34 | CKAP4 | na |  |  | 5527 | 0.549 | 0.0571 | No |
| 35 | TNFRSF1B | na |  |  | 5672 | 0.529 | 0.0547 | No |
| 36 | IGF1R | na |  |  | 6443 | 0.424 | 0.0184 | No |
| 37 | IL3RA | na |  |  | 6563 | 0.408 | 0.0161 | No |
| 38 | NFKBIZ | na |  |  | 6595 | 0.404 | 0.0184 | No |
| 39 | S100A1 | na |  |  | 6693 | 0.394 | 0.0172 | No |
| 40 | CXCL10 | na |  |  | 6890 | 0.372 | 0.0105 | No |
| 41 | TTC39B | na |  |  | 7078 | 0.351 | 0.0041 | No |
| 42 | FGL2 | na |  |  | 7089 | 0.349 | 0.0070 | No |
| 43 | LTB | na |  |  | 7357 | 0.318 | -0.0039 | No |
| 44 | SPRY4 | na |  |  | 7439 | 0.314 | -0.0051 | No |
| 45 | BCL2 | na |  |  | 7623 | 0.289 | -0.0119 | No |
| 46 | ST3GAL4 | na |  |  | 7626 | 0.288 | -0.0092 | No |
| 47 | CDKN1C | na |  |  | 7826 | 0.269 | -0.0170 | No |
| 48 | IRF6 | na |  |  | 8187 | 0.235 | -0.0336 | No |
| 49 | PIM1 | na |  |  | 8219 | 0.229 | -0.0330 | No |
| 50 | TRAF1 | na |  |  | 8359 | 0.212 | -0.0382 | No |
| 51 | CDC6 | na |  |  | 8605 | 0.191 | -0.0492 | No |
| 52 | ODC1 | na |  |  | 8852 | 0.159 | -0.0606 | No |
| 53 | PRKCH | na |  |  | 8871 | 0.156 | -0.0600 | No |
| 54 | IL4R | na |  |  | 9138 | 0.135 | -0.0727 | No |
| 55 | TNFRSF21 | na |  |  | 9191 | 0.127 | -0.0742 | No |
| 56 | RHOB | na |  |  | 9250 | 0.124 | -0.0760 | No |
| 57 | CCNE1 | na |  |  | 9260 | 0.122 | -0.0753 | No |
| 58 | SLC39A8 | na |  |  | 9300 | 0.117 | -0.0762 | No |
| 59 | SOCS1 | na |  |  | 9324 | 0.113 | -0.0763 | No |
| 60 | SCN9A | na |  |  | 9347 | 0.111 | -0.0764 | No |
| 61 | CAPN3 | na |  |  | 9564 | 0.093 | -0.0868 | No |
| 62 | CCND2 | na |  |  | 9609 | 0.088 | -0.0883 | No |
| 63 | XBP1 | na |  |  | 9718 | 0.073 | -0.0932 | No |
| 64 | DHRS3 | na |  |  | 9773 | 0.068 | -0.0954 | No |
| 65 | IGF2R | na |  |  | 10025 | 0.038 | -0.1082 | No |
| 66 | ETV4 | na |  |  | 10161 | 0.020 | -0.1151 | No |
| 67 | COL6A1 | na |  |  | 10352 | 0.012 | -0.1250 | No |
| 68 | BATF | na |  |  | 10429 | 0.004 | -0.1289 | No |
| 69 | ALCAM | na |  |  | 10706 | -0.030 | -0.1431 | No |
| 70 | RABGAP1L | na |  |  | 10753 | -0.033 | -0.1452 | No |
| 71 | SYT11 | na |  |  | 10876 | -0.050 | -0.1511 | No |
| 72 | P2RX4 | na |  |  | 10928 | -0.055 | -0.1533 | No |
| 73 | SOCS2 | na |  |  | 10977 | -0.063 | -0.1552 | No |
| 74 | CD44 | na |  |  | 11081 | -0.075 | -0.1599 | No |
| 75 | DCPS | na |  |  | 11151 | -0.086 | -0.1626 | No |
| 76 | ITGAE | na |  |  | 11419 | -0.104 | -0.1756 | No |
| 77 | CCND3 | na |  |  | 11490 | -0.109 | -0.1783 | No |
| 78 | CD81 | na |  |  | 11515 | -0.114 | -0.1784 | No |
| 79 | ADAM19 | na |  |  | 12139 | -0.191 | -0.2093 | No |
| 80 | TNFRSF9 | na |  |  | 12175 | -0.197 | -0.2092 | No |
| 81 | MYO1E | na |  |  | 12231 | -0.204 | -0.2101 | No |
| 82 | AHCY | na |  |  | 12587 | -0.232 | -0.2264 | No |
| 83 | PRAF2 | na |  |  | 12589 | -0.232 | -0.2242 | No |
| 84 | AHR | na |  |  | 12759 | -0.253 | -0.2306 | No |
| 85 | UMPS | na |  |  | 12776 | -0.255 | -0.2290 | No |
| 86 | FAM126B | na |  |  | 12812 | -0.259 | -0.2283 | No |
| 87 | PDCD2L | na |  |  | 12831 | -0.262 | -0.2267 | No |
| 88 | KLF6 | na |  |  | 12855 | -0.265 | -0.2253 | No |
| 89 | GBP4 | na |  |  | 12911 | -0.273 | -0.2255 | No |
| 90 | SPRED2 | na |  |  | 13004 | -0.284 | -0.2276 | No |
| 91 | CTSZ | na |  |  | 13008 | -0.284 | -0.2250 | No |
| 92 | CSF2 | na |  |  | 13029 | -0.288 | -0.2232 | No |
| 93 | SH3BGRL2 | na |  |  | 13191 | -0.311 | -0.2286 | No |
| 94 | POU2F1 | na |  |  | 13365 | -0.332 | -0.2345 | No |
| 95 | CYFIP1 | na |  |  | 13440 | -0.343 | -0.2350 | No |
| 96 | MXD1 | na |  |  | 13509 | -0.354 | -0.2351 | No |
| 97 | BHLHE40 | na |  |  | 13518 | -0.356 | -0.2321 | No |
| 98 | NRP1 | na |  |  | 13560 | -0.362 | -0.2307 | No |
| 99 | SNX9 | na |  |  | 13607 | -0.371 | -0.2295 | No |
| 100 | CDCP1 | na |  |  | 13699 | -0.386 | -0.2305 | No |
| 101 | ECM1 | na |  |  | 13763 | -0.397 | -0.2299 | No |
| 102 | TGM2 | na |  |  | 13799 | -0.403 | -0.2278 | No |
| 103 | EOMES | na |  |  | 14026 | -0.420 | -0.2356 | No |
| 104 | NCOA3 | na |  |  | 14080 | -0.428 | -0.2342 | No |
| 105 | SNX14 | na |  |  | 14134 | -0.432 | -0.2328 | No |
| 106 | SYNGR2 | na |  |  | 14179 | -0.438 | -0.2308 | No |
| 107 | GPX4 | na |  |  | 14284 | -0.460 | -0.2318 | No |
| 108 | GLIPR2 | na |  |  | 14417 | -0.479 | -0.2340 | No |
| 109 | PTGER2 | na |  |  | 14456 | -0.483 | -0.2313 | No |
| 110 | F2RL2 | na |  |  | 14504 | -0.491 | -0.2290 | No |
| 111 | GUCY1B3 | na |  |  | 14538 | -0.498 | -0.2259 | No |
| 112 | GALM | na |  |  | 14564 | -0.500 | -0.2223 | No |
| 113 | BCL2L1 | na |  |  | 14898 | -0.549 | -0.2344 | No |
| 114 | PHLDA1 | na |  |  | 14990 | -0.561 | -0.2337 | No |
| 115 | TIAM1 | na |  |  | 14991 | -0.561 | -0.2283 | No |
| 116 | PTRH2 | na |  |  | 15433 | -0.638 | -0.2452 | No |
| 117 | CAPG | na |  |  | 15558 | -0.662 | -0.2452 | Yes |
| 118 | CISH | na |  |  | 15622 | -0.672 | -0.2420 | Yes |
| 119 | HK2 | na |  |  | 15767 | -0.695 | -0.2428 | Yes |
| 120 | GABARAPL1 | na |  |  | 15826 | -0.709 | -0.2389 | Yes |
| 121 | PHTF2 | na |  |  | 15865 | -0.717 | -0.2339 | Yes |
| 122 | SERPINB6 | na |  |  | 15888 | -0.721 | -0.2280 | Yes |
| 123 | IFITM3 | na |  |  | 15996 | -0.744 | -0.2264 | Yes |
| 124 | NFIL3 | na |  |  | 16230 | -0.795 | -0.2308 | Yes |
| 125 | PNP | na |  |  | 16242 | -0.798 | -0.2236 | Yes |
| 126 | IKZF2 | na |  |  | 16391 | -0.830 | -0.2233 | Yes |
| 127 | LRRC8C | na |  |  | 16404 | -0.833 | -0.2158 | Yes |
| 128 | P4HA1 | na |  |  | 16447 | -0.842 | -0.2098 | Yes |
| 129 | TWSG1 | na |  |  | 16529 | -0.860 | -0.2056 | Yes |
| 130 | TNFSF10 | na |  |  | 16587 | -0.869 | -0.2002 | Yes |
| 131 | WLS | na |  |  | 16759 | -0.916 | -0.2002 | Yes |
| 132 | SLC2A3 | na |  |  | 16869 | -0.946 | -0.1967 | Yes |
| 133 | ITGAV | na |  |  | 16980 | -0.980 | -0.1929 | Yes |
| 134 | CST7 | na |  |  | 16988 | -0.983 | -0.1837 | Yes |
| 135 | SWAP70 | na |  |  | 17026 | -0.994 | -0.1759 | Yes |
| 136 | ARL4A | na |  |  | 17123 | -1.023 | -0.1710 | Yes |
| 137 | RRAGD | na |  |  | 17307 | -1.086 | -0.1700 | Yes |
| 138 | AMACR | na |  |  | 17354 | -1.102 | -0.1616 | Yes |
| 139 | BMPR2 | na |  |  | 17365 | -1.107 | -0.1514 | Yes |
| 140 | PLSCR1 | na |  |  | 17416 | -1.120 | -0.1430 | Yes |
| 141 | PLIN2 | na |  |  | 17478 | -1.144 | -0.1351 | Yes |
| 142 | NDRG1 | na |  |  | 17623 | -1.208 | -0.1309 | Yes |
| 143 | SMPDL3A | na |  |  | 17743 | -1.259 | -0.1248 | Yes |
| 144 | ANXA4 | na |  |  | 17877 | -1.320 | -0.1189 | Yes |
| 145 | LCLAT1 | na |  |  | 17980 | -1.368 | -0.1109 | Yes |
| 146 | RORA | na |  |  | 18188 | -1.490 | -0.1072 | Yes |
| 147 | PRNP | na |  |  | 18243 | -1.523 | -0.0952 | Yes |
| 148 | NT5E | na |  |  | 18421 | -1.652 | -0.0884 | Yes |
| 149 | ITGA6 | na |  |  | 18471 | -1.687 | -0.0745 | Yes |
| 150 | IFNGR1 | na |  |  | 18491 | -1.701 | -0.0589 | Yes |
| 151 | CDC42SE2 | na |  |  | 18623 | -1.810 | -0.0481 | Yes |
| 152 | CASP3 | na |  |  | 18683 | -1.879 | -0.0329 | Yes |
| 153 | EMP1 | na |  |  | 18689 | -1.884 | -0.0147 | Yes |
| 154 | ENPP1 | na |  |  | 18814 | -2.037 | -0.0014 | Yes |
| 155 | GSTO1 | na |  |  | 18916 | -2.201 | 0.0148 | Yes |
Table: GSEA details [plain text format]

  

Fig 2: HALLMARK\_IL2\_STAT5\_SIGNALING      
 Blue-Pink O' Gram in the Space of the Analyzed GeneSet

  

Fig 3: HALLMARK\_IL2\_STAT5\_SIGNALING: Random ES distribution      
 Gene set null distribution of ES for **HALLMARK\_IL2\_STAT5\_SIGNALING**
